# Detection of circulating carcinoma cells by telomerase activity

**DOI:** 10.1054/bjoc.2000.1662

**Published:** 2001-03

**Authors:** L R Gauthier, C Granotier, J-C Soria, S Faivre, V Boige, E Raymond, F D Boussin

**Affiliations:** Laboratoire de RadioPathologie, DRR/DSV CEA BP6, Fontenay-aux-Roses, 92265, France; Département de Médecine, Institut Gustave Roussy,, Villejuif, 94805, France

**Keywords:** circulating tumour cells, carcinoma, telomerase

## Abstract

Telomerase has been shown to be a marker of epithelial cancer cells. We developed a method that allows the detection of circulating carcinoma cells in the blood of cancer patients. Circulating epithelial cells are harvested from peripheral blood mononuclear cells by immunomagnetic separation using BerEP4-coated beads. A telomeric repeat amplification protocol (TRAP)-ELISA is then used to measure telomerase in harvested epithelial cells. This method is specific and sensitive as demonstrated by experiments using BerEP4-positive and negative cell lines. Whereas we never found telomerase activity in harvested epithelial cells (HEC) samples from 30/30 healthy donors, we have detected telomerase activity in HEC from 11/15 (73%) patients with stage IIIB or IV non-small cell lung cancer (NSCLC) patients and from 8/11 (72%) stage C or D (Dukes classification) colon cancer patients. This non-invasive method could be of great value as a diagnostic or prognostic marker, or for monitoring cancer progression. © 2001 Cancer Research Campaign http://www.bjcancer.com


*British Journal of Cancer* (2001) **84**(5), 631–635
© 2001 Cancer Research Campaign
doi: 10.1054/ bjoc.2000.1662, available online at http://www.idealibrary.com on IDEAL

### Detection of circulating carcinoma cells by telomerase activity

**LR Gauthier[1], C Granotier[1], J-C Soria[2], S Faivre[2], V Boige[2], E Raymond[2] and FD Boussin[1]**

[1]Laboratoire de RadioPathologie, DRR/DSV, CEA, BP6, 92265 Fontenay-aux-Roses, France; [2]Institut Gustave Roussy, Département de Médecine, 94805 Villejuif, France

**Summary** Telomerase has been shown to be a marker of epithelial cancer cells. We developed a method that allows the detection of circulating carcinoma cells in the blood of cancer patients. Circulating epithelial cells are harvested from peripheral blood mononuclear cells by immunomagnetic separation using BerEP4-coated beads. A telomeric repeat amplification protocol (TRAP)-ELISA is then used to measure telomerase in harvested epithelial cells. This method is specific and sensitive as demonstrated by experiments using BerEP4-positive and negative cell lines. Whereas we never found telomerase activity in harvested epithelial cells (HEC) samples from 30/30 healthy donors, we have detected telomerase activity in HEC from 11/15 (73%) patients with stage IIIB or IV non-small cell lung cancer (NSCLC) patients and from 8/11 (72%) stage C or D (Dukes classification) colon cancer patients. This non-invasive method could be of great value as a diagnostic or prognostic marker, or for monitoring cancer progression. © 2001 Cancer Research Campaign http://www.bjcancer.com

**Keywords**: circulating tumour cells; carcinoma; telomerase

Detection of circulating tumour cells and micrometastases in cancer patients could be useful in determining prognosis and in monitoring systemic therapies (Hardingham et al, 1995; Diel et al, 1996; Pantel et al, 1996). Various techniques have been described using conventional cytology, immunochemistry, polymerase chain reaction (PCR) or reverse transcriptase-PCR (RT-PCR), but most of them have limited sensitivity and/or specificity. PCR and RT-PCR have been shown to be of great interest for the detection of chromosomal alteration specific of some haematological malignancies or non-epithelial tumours. Methods, which have been developed for the detection of epithelium-derived cancer cells are primarily based on the amplification of tissue or lineage specific genes (Bostick et al, 1998; Ghossein et al, 1998; Jung et al, 1999). Therefore, due in part to the occurrence of circulating normal epithelial cells, they fail to be specific for the detection of circulating carcinoma cell.

Telomerase, a specialized reverse trancriptase that adds telomeric repeats at chromosome ends, is a potentially important biomarker and prognostic indicator of cancer (Albanell et al, 1997; Engelhardt et al, 1997). Absent from most of normal somatic cells, with the exception of germ cells, stem cells and activated haematopoietic cells (Hiyama et al, 1995), telomerase is expressed in most tumour cell lines and in a majority of cancer. Reactivation of telomerase activity by cancer cells appears to be an important step in tumorigenesis.

We aimed to assess the feasibility of the use of telomerase detection for the identification of circulating epithelial tumor cells in the blood of cancer patients. Because of the normal expression of telomerase activity by activated lymphocytes (Broccoli et al, 1995; Hiyama et al, 1995) in the blood, we developed a method that combines an immunomagnetic isolation step allowing the separation of epithelial cells from blood cells prior to perform the measurement of telomerase activity by a telomeric repeat amplification protocol (TRAP)-ELISA. This method has been previously applied to stage IV breast cancer patients, showing that 21/25 of them were positive (Soria et al, 1999). Detection of telomerase in circulating epithelial cells could thus represent an important predictive tool to evaluate prognosis and to monitor therapy in patients with cancer. Here, we demonstrate that this technique is sensitive and specific and could be applied to other carcinoma patients with metastatic lung and colon cancer.

*Received 10 August 2000*
*Revised 20 November 2000*
*Accepted 29 November 2000*

*Correspondence to:* FD Boussin

### MATERIALS AND METHODS

### Isolation of circulating epithelial cells

Blood samples (10 ml) were collected in heparinized tubes from 15 patients with stage IIIB ($n$ = 2) or IV ($n$ = 13) non-small cell lung cancer (NSCLC), ages 38 to 71, 11 patients with colon cancer (stage C ($n$ = 2) or D ($n$ = 9), Dukes classification), ages 28 to 83, and 30 age-matched healthy volunteers. Blood samples were stored at room temperature for a maximum of 2 hours before experiments. Peripheral blood mononuclear cells (PBMC) were then separated by Ficoll density gradient (Figure 1A). Briefly blood samples were diluted in 3 volumes of RPMI, layered on 10 ml of Ficoll (Life Technologies, Cergy Pontoise, France) and then centrifuged at 2200 rpm for 25 min at 20˚C. PBMC were then collected, washed 2 times in RPMI, and then resuspended in 1 ml of PBS, 2% fetal calf serum (FCS). Then, $12.5 \times 10^6$ prewashed immunomagnetic beads covalently coated with the BerEP4 monoclonal antibody (DYNAL A.S., Oslo, Norway) were added (Figure 1B). The BerEP4 monoclonal antibody recognizes an epitope on the protein moiety of two 34 and 39 kDa glycopeptides expressed at the surface of epithelial cells in normal and malignant tissues (Latza et al, 1990). After 30 min at 4˚C, cells bound to the beads were harvested using a magnetic field (Figure 1C). Harvested epithelial cells (HEC) were then washed 3 times with PBS, 2% FCS. Washing efficiency was systematically controlled by microscopic examination (Figure 2). Immunofluorescence staining performed on some selected samples using monoclonal antibodies directed against lymphocyte determinants:

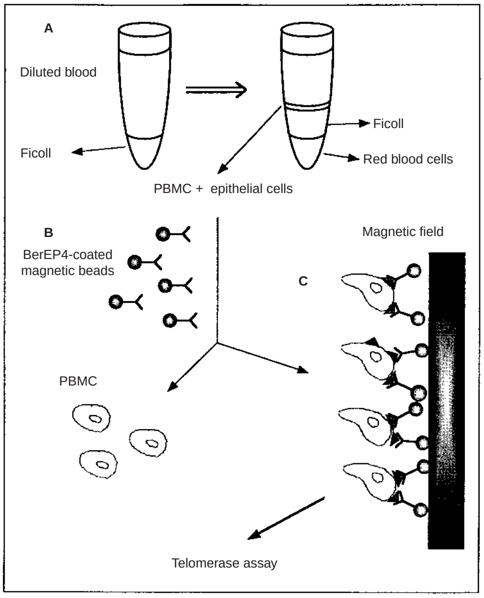

**Figure 1** Method of detection of telomerase activity in circulating epithelial cells. Blood samples are first subjected to Ficoll density gradient. Circulating epithelial cells are then harvested from peripheral blood mononuclear cells (PBMC) by immunomagnetic separation using BerEP4-coated beads. Finally, telomerase activity is assayed on BerEP4 positive-circulating epithelial cells

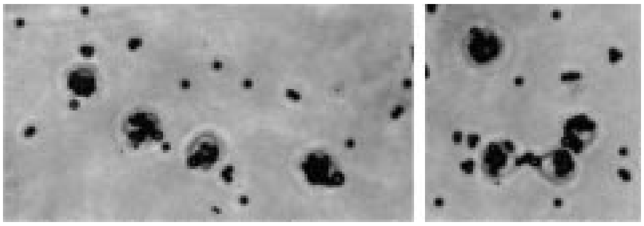

**Figure 2** Circulating epithelial cells collected using BerEP4-coated immunomagnetic beads from a stage IV NSCLC patient. Note the size of the BerEP4-positive cells compared to the beads immunomagnetic beads (2.8 μm)

CD3 (Becton Dickinson) and CD45 (DAKO), have confirmed the lack of detectable contaminating lymphocytes among HEC fractions (data not shown). HEC were incubated for 30 min at 4˚C in 100 μl of lysis buffer (CHAPS) containing 150 U $ml^{-1}$ RNasin (Promega, Madison, WI, USA). After a centrifugation at 16 000 ***g*** for 20 min at 4˚C, supernatants were collected and stored at –80˚C until telomerase activity assay was performed. Protein concentrations were determined using the Bio-Rad Protein Assay (Bio-Rad Laboratories, California).

To evaluate the sensitivity and the specificity of the assay, decreasing number of telomerase-positive cancer cells were added to PBMC samples obtained from an healthy volunteer by serial dilutions, and then submitted to immunomagnetic separation as described previously. We used two human BerEP4-expressing carcinoma cell lines: KB3.1, MCF-7 (Latza et al, 1990) and one human BerEP4-negative leukaemia cell line: Kit 225 (kindly provided by A Vazquez, INSERM U131, Clamart, France). We have previously confirmed that KB3.1 and MCF7 cells express BerEP4, although, mean number of immunomagnetic beads coated on cells tended to indicate that KB3.1 cells express significantly lower levels of BerEP4 than MCF7 do (data not shown). Experiments were repeated at least twice for each cell line.

### Telomeric repeat amplification protocol (TRAP)

Telomerase activity was measured twice in independent experiments on 1 μg of proteins or on 20 μl of cell extracts when insufficient amounts of proteins were available. Furthermore, because telomerase has an essential RNA component, aliquots of samples were treated with RNase to assess the specificity of the reaction (Kim et al, 1994). Assays were performed using TRAPeze ELISA Telomerase detection kit (Oncor, Gaitherburg, MD) according to manufacturer's instructions. In brief, cell extracts were incubated with biotinylated telomerase substrate oligonucleotide (b-TS) at 30˚C for 30 min. The extended products were amplified by a polymerase chain reaction (PCR) using Taq polymerase (Pharmacia Biotech, Uppsala, Sweden), the b-TS, RP primers and a deoxynucleotide mix containing dCTP labeled with dinitrophenyl (DNP). The PCR conditions were 33 cycles of 94˚C for 30 s and 55˚C for 30 s on a PTC-200 thermocycler (M.J. Research, Watertown, MA). The amplification products were immobilized onto streptavidin-coated microtitre plates via biotin-streptavidin interaction, and then detected by anti-DNP antibody conjugated to horseradish peroxidase (HPR). After the addition of the peroxidase substrate (3,3′,5,5′-tetramethylbenzidine or TMB), the amount of TRAP products was determined by the measurement of the absorbance at 450 nm and 690 nm.

Moreover, to confirm the ELISA results, amplified products were systematically run on a 12.5% non-denaturing polyacrylamide gel electrophoresis (PAGE) in 0.5X TBE. Gels were stained with SYBRgreen (dilution 1/10 000) and PCR amplification products were visualized under UV light exposition. Because samples could contain *Taq* polymerase inhibitors, an

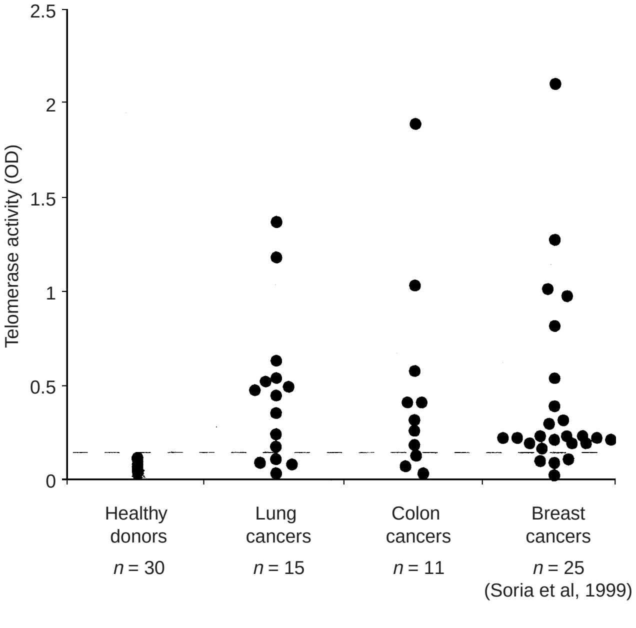

**Figure 3** Telomerase detection by TRAP-ELISA assay in HEC from lung, colon and breast cancers (Soria et al, 1999). The cut-off of positivity was fixed at 0.150, as recommended by the manufacturer and is represented by the dotted line

 © 2001 Cancer Research Campaign internal control band of 36 bp should be visible in all lanes on PAGE.

### RESULTS

Telomerase activity was never detected in the HEC fractions collected from 30 healthy donors (Figure 3), whereas telomerase was present in most of the corresponding BerEP4-negative fractions (data not shown). This is consistent with previous data showing that activated lymphocytes express telomerase (Broccoli et al, 1995; Hiyama et al, 1995).

In order to develop a model system for the detection of rare telomerase-positive epithelial cells in blood of cancer patients, various numbers of cultured BerEP4-expressing carcinoma cells (KB3.1 and MCF-7) were seeded into PBMC isolated from a healthy donor. After immunomagnetic separation, we found

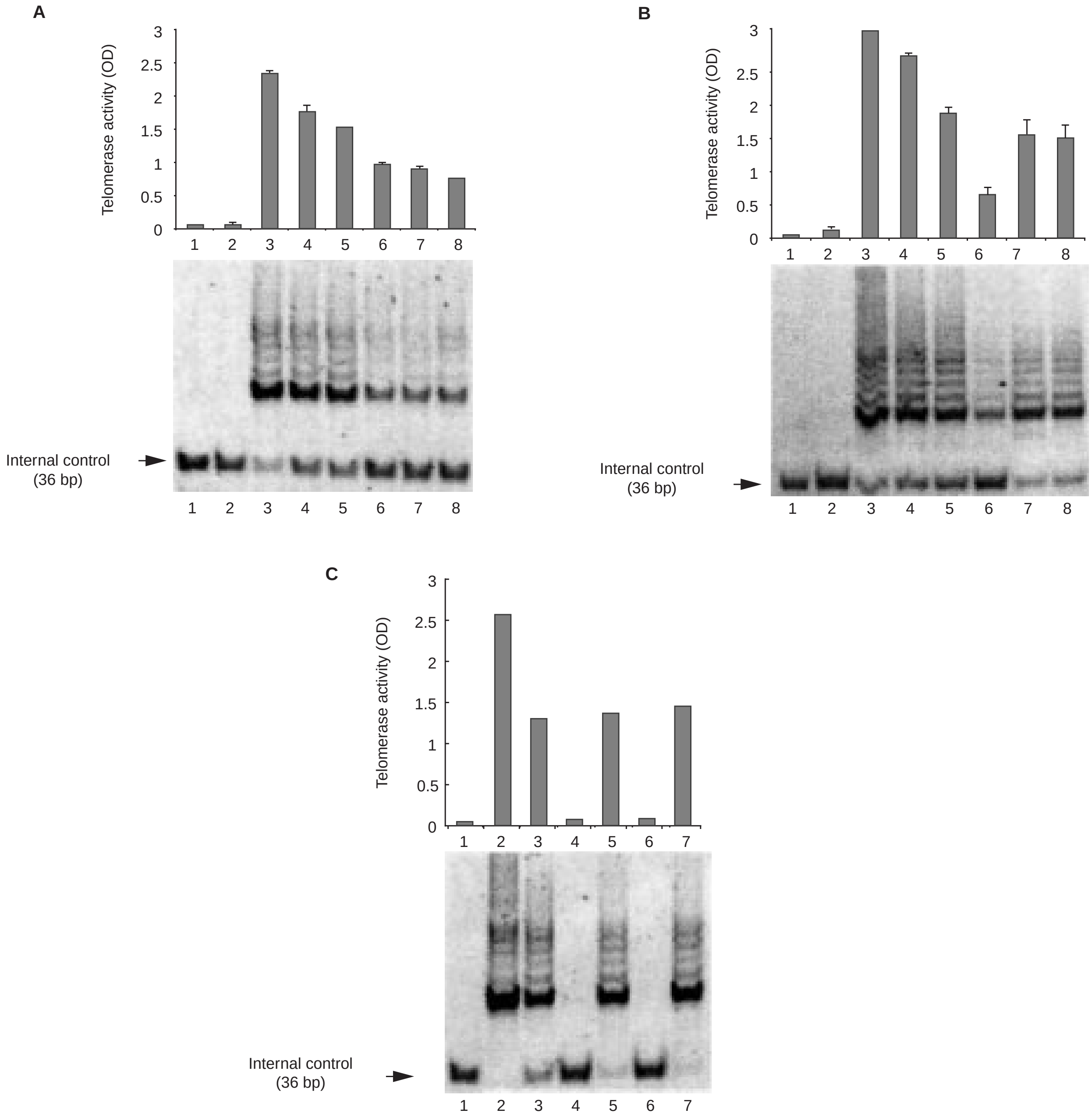

**Figure 4** Sensitivity and specificity of telomerase detection in BerEP4-bearing cells harvested from PBMC. The figures (**A**) and (**B**) show the results of typical experiments in which KB31 (**A**) and MCF7 (**B**) were serially diluted in PBMC obtained from healthy donors and then submitted to immunomagnetic separation using BerEP4-coated beads, as described in the Materials and Methods section. ELISA results (up) of telomerase activity assays were confirmed by a direct visualization of the TRAP ladder by PAGE and SYBRgreen staining (down). Bars indicate variations between two independent measurements of telomerase activity. Lane 1: negative control (CHAPS); lane 2: HEC obtained from unseeded PBMC samples of the donor; lanes 3–8: 3000, 1000, 333, 111, 37, and 12 KB3.1 (**A**) or MCF7 (**B**) cells, respectively, seeded per PBMC samples isolated from 10 ml of blood. (**C**) Same experiment using the BerEP4-negative leukemia cell line KIT225. Lane 1: negative control (CHAPS); lane 2: KIT 225; lane 3: unseeded PBMC; lane 4: HEC obtained from unseeded PBMC; lane 5: BerEP4-negative fraction after immunomagnetic separation perform on unseeded PBMC; lane 6: HEC obtained from PBMC samples seeded with 3000 KIT 225 cells; lane 7: BerEP4-negative fraction obtained from PBMC samples seeded with 3000 KIT 225 cells

*© 2001 Cancer Research Campaign* 

telomerase activity in BerEP4-positive cell fractions, even when only 12 carcinoma cells were added to PBMC samples isolated from 10 ml of whole blood (Figure 4A and 4B).

No telomerase activity was detected in the HEC fractions when the same experiment was performed with the BerEP4-negative leukaemia cell line Kit 225 that express high levels of telomerase activity (Figure 4C). Altogether, these results suggest that our technique is sensitive and specific and allows the detection of rare telomerase-positive BerEP4-bearing epithelial cells in blood of cancer patients.

We thus applied this protocol to blood samples obtained from cancer patients. We detected telomerase activity in HEC from 11/15 (73%) NSCLC patients and from 8/11 (72%) colon cancer patients (Figure 3). Interestingly, the 2 patients with stage IIIB NSCLC and also the 2 patients with stage C colon cancer were telomerase-positive. RNase treatment of cell extracts prior to telomerase assay completely eliminated the signals demonstrating the specificity of the enzymatic detection. Furthermore, telomerase positive samples showed the characteristic processive 6-base pair ladder upon polyacrylamide gel electrophoresis (data not shown).

### DISCUSSION

Here we have demonstrated that our method allows the specific and sensitive detection of circulating telomerase-positive epithelial cells in the blood of carcinoma patients. The normal expression of telomerase by activated lymphocytes does not allow a direct measure of telomerase activity in blood samples. To overcome this problem, we used BerEP4-coated magnetic beads to harvest epithelial cells from blood samples. In preliminary experiments, we found that better results were obtained in doing this purification step on PBMC isolated by Ficoll gradient centrifugation. Microscope examination and immunofluorescence staining have shown the lack of detectable lymphocytes in HEC samples obtained by this technique. The specificity of the overall method was further confirmed by the lack of detectable telomerase activity in HEC obtained from healthy donors, even when cells from the highly telomerase-positive leukaemia cell line KIT 225 were added to PBMC samples prior to immunomagnetic separation.

Experiments using two carcinoma cell lines revealed that our method allows the detection of nearly 12 telomerase-positive BerEP4-bearing cells/PBMC isolated from 10 ml whole blood. Although levels of telomerase activity could differ considerably between primary cancer cells and cultured cell lines, this result shows that our method is adapted for the detection of extremely rare circulating carcinoma cells.

Telomerase has been shown to be a marker of epithelial cancer cells. We have previously detected telomerase activity in circulating epithelial cells in 84% of metastatic breast cancer patients (Soria et al, 1999). We have shown here that our method could be successfully applied to other carcinomas such as colon and lung cancers. Whereas normal colonic crypt epithelial cells exhibiting stem cells properties express very weak telomerase activity within the proliferative zone of the crypt (Tahara et al, 1995; Kolquist et al, 1998), evidences have been accumulated over years to suggest that telomerase is up-regulated as a function of increased tumour cell invasion, tumour progression, and metastatic potential in colon cancer (Engelhardt et al, 1997; Yoshida et al, 1999). Similarly, at the tumour site, telomerase activity has been shown to be an important prognostic factor for NSCLC (Albanell et al, 1997; Marchetti et al, 1999; Taga et al, 1999). Whereas we never found telomerase activity in HEC samples from 30/30 healthy donors, telomerase was expressed in more than 72% of the patients with advanced carcinoma involved in this study. Including our previous data concerning breast cancer patients (Soria et al, 1999), we therefore found telomerase activity in HEC from 40/51 (78%) patients with advanced cancer. Further studies, involving particularly patients at different stages of the disease are now required to determine the precise clinical and biological significance of telomerase expression or its lack in HEC. However the fact that we found telomerase activity in HEC from 2/2 patients with stage IIIB NSCLC and also 2/2 patients with stage C colon cancer highly suggest that our method could be used at earlier stages of cancer progression.

To conclude we have described here a simple and reliable method based on telomerase activity assays that allows a specific and sensitive detection of circulating carcinoma cells. Telomerase detection on HEC may be more specific compared to other described techniques involving epithelial specific genes (Bostick et al, 1998; Ghossein et al, 1998; Jung et al, 1999) because it allows a direct detection of a marker of epithelial tumour cells. However, further studies should be performed to demonstrate the value of this non-invasive method in determining diagnostic, prognostic and response to therapy.

### ACKNOWLEDGEMENTS

The authors thank the nursing staff of the Service Gard in the département de Médecine at Institut Gustave Roussy for their helpful contributions.

### REFERENCES

Albanell J, Lonardo F, Rusch V, Engelhardt M, Langenfeld J, Han W, Klimstra D, Venkatraman E, Moore MA and Dmitrovsky E (1997) High telomerase activity in primary lung cancers: association with increased cell proliferation rates and advanced pathologic stage. *J Natl Cancer Inst* **89**: 1609–1615

Bostick PJ, Chatterjee S, Chi DD, Huynh KT, Giuliano AE, Cote R and Hoon DS (1998) Limitations of specific reverse-transcriptase polymerase chain reaction markers in the detection of metastases in the lymph nodes and blood of breast cancer patients. *J Clin Oncol* **16**: 2632–2640

Broccoli D, Young JW and de Lange T (1995) Telomerase activity in normal and malignant hematopoietic cells. *Proc Natl Acad Sci USA* **92**: 9082–9086

Diel IJ, Kaufmann M, Costa SD, Holle R, von Minckwitz G, Solomayer EF, Kaul S and Bastert G (1996) Micrometastatic breast cancer cells in bone marrow at primary surgery: prognostic value in comparison with nodal status [see comments]. *J Natl Cancer Inst* **88**: 1652–1658

Engelhardt M, Drullinsky P, Guillem J and Moore MA (1997) Telomerase and telomere length in the development and progression of premalignant lesions to colorectal cancer. *Clin Cancer Res* **3**: 1931–1941

Ghossein RA, Coit D, Brennan M, Zhang ZF, Wang Y, Bhattacharya S, Houghton A and Rosai J (1998) Prognostic significance of peripheral blood and bone marrow tyrosinase messenger RNA in malignant melanoma. *Clin Cancer Res* **4**: 419–428

Hardingham JE, Kotasek D, Sage RE, Eaton MC, Pascoe VH and Dobrovic A (1995) Detection of circulating tumor cells in colorectal cancer by immunobead-PCR is a sensitive prognostic marker for relapse of disease. *Mol Med* **1**: 789–794

Hiyama K, Hirai Y, Kyoizumi S, Akiyama M, Hiyama E, Piatyszek MA, Shay JW, Ishioka S and Yamakido M (1995) Activation of telomerase in human lymphocytes and hematopoietic progenitor cells. *J Immunol* **155**: 3711–3715

Jung R, Petersen K, Kruger W, Wolf M, Wagener C, Zander A and Neumaier M (1999) Detection of micrometastasis by cytokeratin 20 RT-PCR is limited due to stable background transcription in granulocytes. *Br J Cancer* **81**: 870–873

© 2001 Cancer Research Campaign

Kim NW, Piatyszek MA, Prowse KR, Harley CB, West MD, Ho PL, Coviello GM, Wright WE, Weinrich SL and Shay JW (1994) Specific association of human telomerase activity with immortal cells and cancer [see comments]. *Science* **266**: 2011–2015

Kolquist KA, Ellisen LW, Counter CM, Meyerson M, Tan LK, Weinberg RA, Haber DA and Gerald WL (1998) Expression of TERT in early premalignant lesions and a subset of cells in normal tissues [see comments]. *Nat Genet* **19**: 182–186

Latza U, Niedobitek G, Schwarting R, Nekarda H and Stein H (1990) Ber-EP4: new monoclonal antibody which distinguishes epithelia from mesothelial. *J Clin Pathol* **43**: 213–219

Marchetti A, Bertacca G, Buttitta F, Chella A, Quattrocolo G, Angeletti CA and Bevilacqua G (1999) Telomerase activity as a prognostic indicator in stage I non-small cell lung cancer. *Clin Cancer Res* **5**: 2077–2081

Pantel K, Izbicki J, Passlick B, Angstwurm M, Haussinger K, Thetter O and Riethmuller G (1996) Frequency and prognostic significance of isolated tumour cells in bone marrow of patients with non-small-cell lung cancer without overt metastases. *Lancet* **347**: 649–653

Soria JC, Gauthier LR, Raymond E, Granotier C, Morat L, Armand JP, Boussin FD and Sabatier L (1999) Molecular detection of telomerase-positive circulating epithelial cells in metastatic breast cancer patients. *Clin Cancer Res* **5**: 971–975

Taga S, Osaki T, Ohgami A, Imoto H and Yasumoto K (1999) Prognostic impact of telomerase activity in non-small cell lung cancers. *Ann Surg* **230**: 715–720

Tahara H, Kuniyasu H, Yokozaki H, Yasui W, Shay JW, Ide T and Tahara E (1995) Telomerase activity in preneoplastic and neoplastic gastric and colorectal lesions. *Clin Cancer Res* **1**: 1245–1251

Yoshida R, Kiyozuka Y, Ichiyoshi H, Senzaki H, Takada H, Hioki K and Tsubura A (1999) Change in telomerase activity during human colorectal carcinogenesis. *Anticancer Res* **19**: 2167–2172

*© 2001 Cancer Research Campaign*
